# Genome-Wide Identification, Characterization, and Expression Analysis of Orphan Genes Within Coriander

**DOI:** 10.3390/plants14050778

**Published:** 2025-03-03

**Authors:** Meidi Zhang, Mo Zhu, Hong Lang, Weiming Wang, Xiaonan Li, Mingliang Jiang

**Affiliations:** 1 School of Agriculture, Jilin Agricultural Science and Technology University, Jilin 132101, China; zhangmeidi@jlnku.edu.cn (M.Z.); langhong@jlnku.edu.cn (H.L.); wangweiming_jlnku@163.com (W.W.); 2 Institute of Agricultural Biotechnology, Jilin Academy of Agricultural Sciences (Northeast Agricultural Research Center of China), Changchun 130033, China; zhumo8989@163.com; 3 Molecular Biology of Vegetable Laboratory, College of Horticulture, Shenyang Agricultural University, Shenyang 110866, China

**Keywords:** coriander, orphan genes, identification, characterization, expression analysis

## Abstract

Orphan genes (*OGs*) are genes that have no significant sequence similarity with known genes from other species or lineages. Identifying and characterizing *OGs* have become more feasible with the increasing availability of plant transcriptomes and genome sequences. *OGs* play important roles in response to both biotic and abiotic stresses, contributing to biological functions and lineage-specific traits. This study aimed to identify and characterize *OGs* in *Coriandrum sativum* (coriander) using the BLAST method. A total of 941 *C. sativum OGs* (*CsOGs*), 1298 Apiaceae-specific genes (*ASGs*), and 38,508 evolutionarily conserved genes (*ECGs*) were identified through comparative genomics. Genic feature analyses revealed that *CsOGs* and *ASGs*, although part of different gene sets, had shorter gene lengths, a lower proportion of multi-exon genes, and higher GC content than *ECGs*. *OGs* were distributed across all 11 chromosomes, with the highest proportion of *CsOGs* and *ASGs* found on chromosome A11. RNA-Seq analysis revealed 71 *CsOGs* uniquely expressed in four different tissues, 61 *CsOGs* specifically expressed across three growth stages, and five *CsOGs* with specific expression patterns in different tissues and growth stages. Notably, as determined via qRT-PCR analysis, these five *CsOGs* presented general or specific expression patterns under normal conditions, but their expression significantly increased after exposure to cold stress, suggesting that they may play a critical role in cold stress response. This study comprehensively identified, characterized, and analyzed the expression of *OGs* within coriander, which provides a foundation for further research on the functions of coriander *OGs* in influencing species-specific trait formation and stress response.

## 1. Introduction

Orphan genes (*OGs*) are defined as genes that have no significant similarity to other sequences in other species [1,2]. Genetic mechanisms contributing to the emergence of *OGs* include gene fusion and fission, gene duplication, retroposition, lateral gene transfer, and exon shuffling [3]. However, gene exaptation and duplication were identified from transposable elements (TEs) as the primary mechanisms for *OG* emergence [4]. *OGs* may evolve from non-coding sequences or derive from older coding sequences. Their emergence and fixation in the genome, along with their participation in regulatory networks, indicate evolutionary maturity [5]. Over 50% of *OGs* in *Arabidopsis thaliana* resemble non-coding regions in *A. lyrata*, suggesting de novo formation [4]. De novo gene creation in a species’ gene pool may be more common than sporadic gene copying and is a primary mechanism of *OG* formation [6,7]. Although the origin and evolution of *OGs* are not fully understood, their emergence provides insights into species evolution, so genome and transcriptome data have been widely used to accelerate *OG* identification [8]. *OGs* have been identified in various species or lineages, including *A. thaliana* [9], *Oryza sativa* [10,11], *Vigna unguiculata* [12], *Aegiceras corniculatum* [13], *Triticeae* [14], and *Brassica rapa* [2]. Phylogenetically, *OGs* participate in species-specific evolution, stress response, and metabolic regulation [2,8,15,16], playing a vital role in species divergence [14]. Most *OG* functions are unknown, likely due to the absence of homologous sequences or identifiable functional domains.

Despite the challenges in studying the functions of *OGs*, some studies have provided insights into their roles. For example, *B. rapa OG BOLTING RESISTANCE 1* (*BR1*) regulates flowering time in *Arabidopsis*, potentially involving vernalization and photoperiodic pathways [17]. The mutant form of *B. rapa OG1* (*BrOG1*) presents increased sucrose synthase (SUS) activity and sucrose content, whereas invertase activity remains unchanged, indicating its role in soluble sugar metabolism [18]. The *Arabidopsis OG Qua-Quine Starch* (*QQS*) regulates carbon and nitrogen allocation in soybean. When *QQS* is transferred to soybean, lines expressing *QQS* show a 60% increase in leaf protein and an 80% decrease in leaf starch [9]. *OG BOLTING RESISTANCE 2* (*BR2*) is associated with bolting resistance, and its overexpression significantly delays flowering in *Arabidopsis* transgenic plants while also affecting leaf and flower development [19]. The Poaceae-specific *Male Sterility 1* (*Ms1*) gene encodes a phospholipid-binding protein involved in male sterility, variation, and pollen outer wall development. It is specifically expressed in microspore cells and plays a vital role in microgametogenesis [20]. Similarly, the *OG Male Sterility 2* (*Ms2*) in grass species induces male sterility in *Brachypodium*, barley, and wheat, with its expression in anthers related to the insertion of a reverse transcription element in the promoter [21]. These findings provide a theoretical basis for the functional identification of *OGs*. However, the functions of these *OGs* remain unidentified in newly sequenced genomes, such as those of *C. sativum*.

Several species from the Apiaceae family are cultivated globally for vegetable or medicinal purposes, including *Apium graveolens* (celery), *C. sativum*, and *Daucus carota* (carrot) [22]. *Coriandrum sativum*, also known as Chinese parsley or coriander, is an important crop used as a spice, fragrance, vegetable, and traditional medicine [23]. Coriander, a well-known herb distributed across North Africa, Central Europe, and other regions, contains bioactive compounds such as polyphenols, phenolic acids, flavonoids, and essential oils. All parts of coriander can be used as seasonings or essential for oil extraction [24,25]. Coriander is widely used in the pharmaceutical and food industries, with recent studies demonstrating its antioxidant, antibacterial, antidiabetic, antimutagenic, and anticancer properties [26,27]. Low-temperature stress affects coriander plant growth, yield components, and metabolic activities [28]. Gene families such as heat shock transcription factors (*Hsf*) and *TEOSINTE BRANCHED 1/CYCLOIDEA/PROLIFERATING CELL FACTOR 1/2* (*TCP*) have been identified and characterized in coriander, facilitating functional and comparative genomics studies [22,24]. Furthermore, interaction networks between pathway-related genes (tyrosine, porphyrins and chlorophyll, and vitamin E metabolic pathways) and transcription factors (TFs) have been constructed, supporting the activation of genes related to the nutritional and medicinal value of coriander [29]. As an important seasoning vegetable, coriander’s flavor, disease resistance, and environmental adaptability are important breeding goals. *OGs* may be involved in regulating these traits, so studying these *OGs* can provide new targets and tools for the molecular breeding of coriander. However, *OGs* have not yet been identified in coriander. Genome sequencing of *C. sativum* [23], *D. carota* [30], and *A. graveolens* [31,32] from the Apiaceae family provides a foundation for screening *OGs* within coriander.

In this study, Apiaceae-specific genes (*ASGs*), *C. sativum OGs* (*CsOGs*), and evolutionary-conserved genes (*ECGs*) were identified and characterized from the coriander genome via stringent homologous sequence criteria compared with previous comparative analyses of *OGs* [2]. To explore *CsOG* functions, published RNA sequencing data [23] and qRT-PCR analysis were used to investigate the expression patterns of *CsOGs* across different tissues, at different growth stages, and under cold stress treatments. This study serves as a valuable resource for further exploration of the biological functions of *OGs* in coriander.

## 2. Results

### 2.1. Identification of CsOGs and ASGs in C. sativum

**Figure 1:**
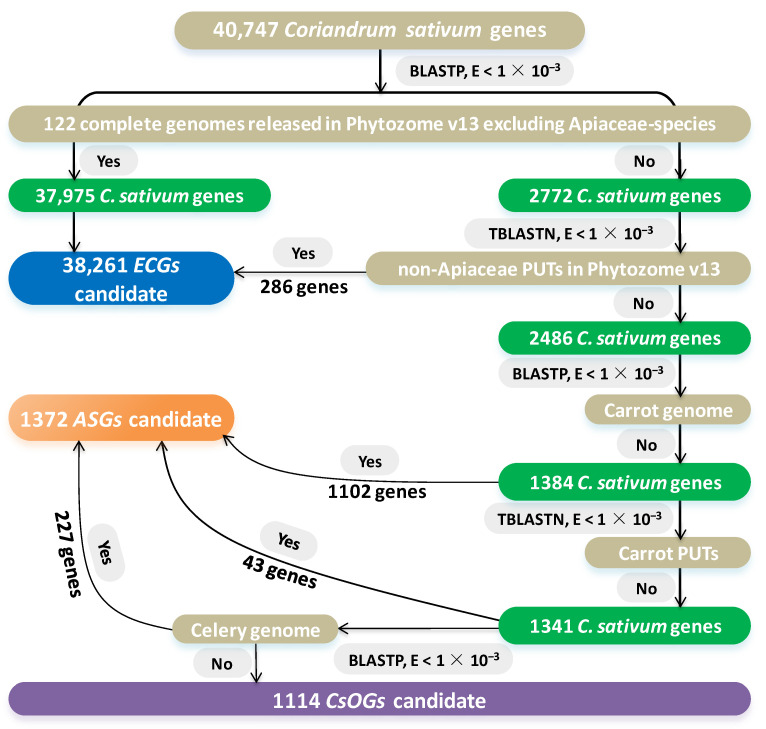
Procedure for identifying candidate *ASGs*, *CsOGs*, and *ECGs* in the coriander genome. The gray boxes represent plant genomes and PlantGDB-assembled unique transcripts (PUTs). The green boxes represent coriander genes. The blue boxes indicate coriander genes identified as *ECG* candidates, whereas the orange and purple boxes represent coriander genes identified as *ASG* and *CsOG* candidates, respectively.

A method of introducing stricter manual inspection incorporating transcriptome and plant genome sequences was employed to screen *ECGs*, *ASGs*, and *CsOGs* [2,33,34,35,36,37]. A total of 40,747 *C. sativum* genes were compared against 122 complete genomes available in Phytozome v13, excluding Apiaceae species, using BLASTP v2.2.31. Among these genes, 37,975 genes showed significant sequence similarity (E-value < 1 × 10^−3^) with at least one species outside the Apiaceae family and were classified as *ECG* candidates; thus, these genes were excluded from further analysis (Figure 1). The remaining 2772 *C. sativum* genes with no significant similarity to non-Apiaceae genomes were compared against non-Apiaceae PlantGDB-generated unique transcripts (PUTs) in Phytozome v13 using TBLASTN. Among them, 286 genes had significant similarity (E-value < 1 × 10^−3^) to at least one non-Apiaceae PUT, classifying them as additional *ECG* candidates and removing them from further analyses. The remaining 2486 *C. sativum* genes presented no significant similarity with non-Apiaceae PUTs. Further comparisons with *Daucus carota* (carrot) identified 1102 genes with significant similarity (E-value < 1 × 10^−3^), which were defined as *ASG* candidates and excluded from further analyses (Figure 1). The remaining 1384 *C. sativum* genes were compared with carrot PUTs using TBLASTN, resulting in 43 genes identified as *ASG* candidates. In the next step, 1341 genes were compared with the celery genome, revealing 227 genes with significant similarity (E-value < 1 × 10^−3^). The remaining 1114 *C. sativum* genes, which lacked significant similarity across all comparisons, were classified as *CsOG* candidates (Figure 1).

**Figure 2:**
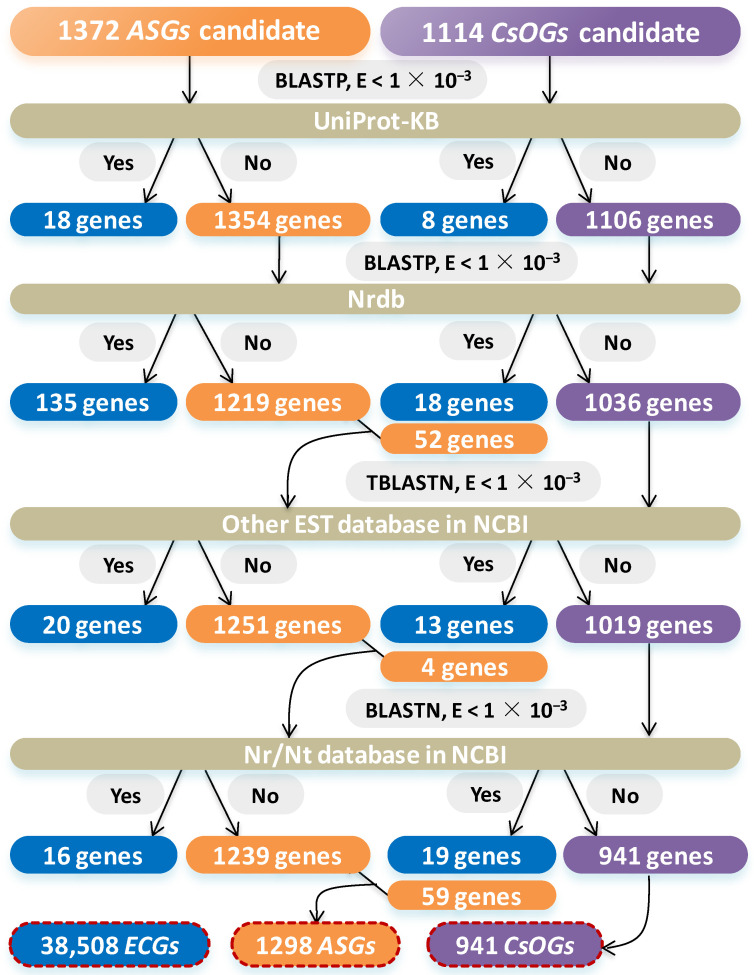
Procedure for identifying *CsOGs*, *ASGs*, and *ECGs* in coriander genome. Nrdb indicates Non-redundant protein database, ESTs represents expressed sequence tags, and Nr/Nt indicates nucleotide collection. The blue, orange, and purple boxes bordered by red dotted lines represent coriander genes identified as *ECGs*, *ASGs*, and *CsOGs*, respectively.

To improve accuracy and minimize false positives, the Non-redundant protein database (Nrdb) and UniProt-KB were used in BLASTP to compare candidate *CsOGs* and *ASGs* (Figure 2). After blast (E-value < 1 × 10^−3^), 26 genes (18 *ASGs* and 8 *CsOGs*) were classified as *ECGs*. BLASTP analysis of 1354 *ASGs* and 1106 *CsOGs* against Nrdb identified 153 genes (135 *ASGs* and 18 *CsOGs*) as *ECGs*, while 52 *CsOGs* with significant similarity (E-value < 1 × 10^−3^) were reclassified as *ASGs*. TBLASTN comparison of 1271 *ASGs* and 1036 *CsOGs* against other EST databases in NCBI identified 33 genes (20 *ASGs* and 13 *CsOGs*) as *ECGs*, with 4 *CsOGs* redefined as *ASGs*. Moreover, BLASTN comparison of 1255 *ASGs* and 1019 *CsOGs* against the Nr/Nt database in NCBI identified 35 genes (16 *ASGs* and 19 *CsOGs*) as *ECGs*, while 59 *CsOGs* showed significant similarity (E-value < 1 × 10^−3^) and were redefined as *ASGs*. In summary, a total of 38,508 *ECGs*, 1298 *ASGs*, and 941 *CsOGs* were identified.

### 2.2. Genic Features of the CsOGs and ASGs in C. sativum

The genic characteristics of the *CsOGs*, *ASGs*, and *ECGs* were compared to determine potential differences (Table 1). The results indicated that the average protein coding sequence (CDS) and gene lengths of the *CsOGs* and *ASGs* were significantly shorter than those of the *ECGs*. Moreover, the average number of exons per gene in the *CsOGs* and *ASGs* was lower than that in the *ECGs*, while the intron lengths in the *CsOGs* and *ASGs* were longer than that in *ECGs*. The GC content of CDS, intron, and whole-gene regions did not significantly differ among the *CsOGs*, *ASGs*, and *ECGs*.

**Figure 3:**
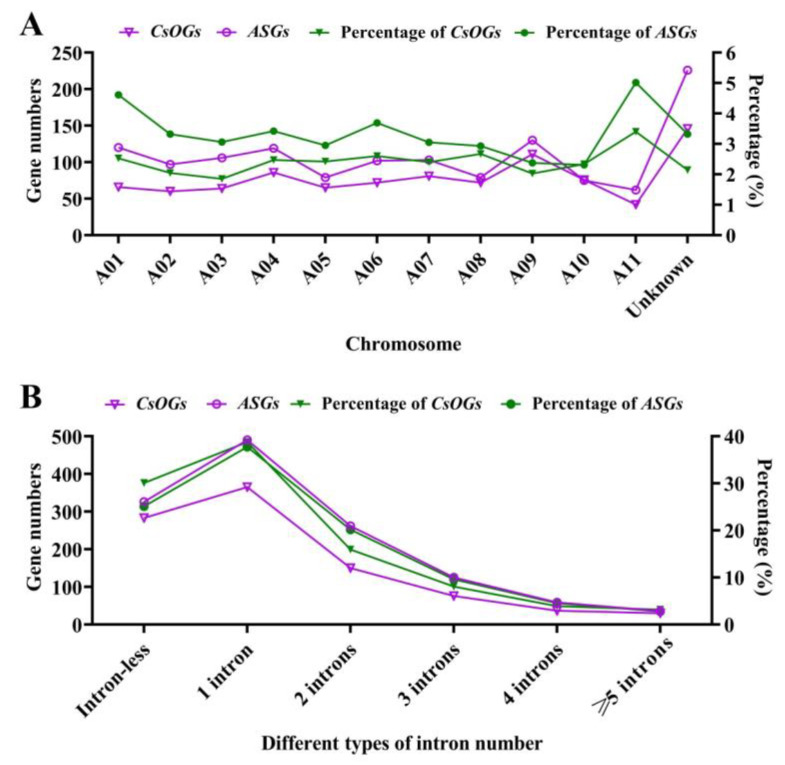
Analysis of the chromosome distribution and intron numbers of *CsOGs* and *ASGs* in coriander. (**A**) Chromosome distribution of *CsOGs* and *ASGs* in coriander, showing both numbers and percentages. The X-axis represents the eleven chromosomes and scaffold (represented by unknown) of the coriander. The left Y-axis indicates the number of *CsOGs* (purple line with triangles) and *ASGs* (purple line with circles) on each chromosome, while the right Y-axis represents the percentage of *CsOGs* (green line with triangles) and *ASGs* (green line with circles) per chromosome. (**B**) Intron number analysis of *CsOGs* and *ASGs*. Intron numbers are classified into six categories, as indicated on the X-axis. The left Y-axis shows the number of *CsOGs* (purple line with triangles) and *ASGs* (purple line with circles) for each intron category, while the right Y-axis represents the percentage of *CsOGs* (green line with triangles) and *ASGs* (green line with circles) for each intron category.

**Figure 4:**
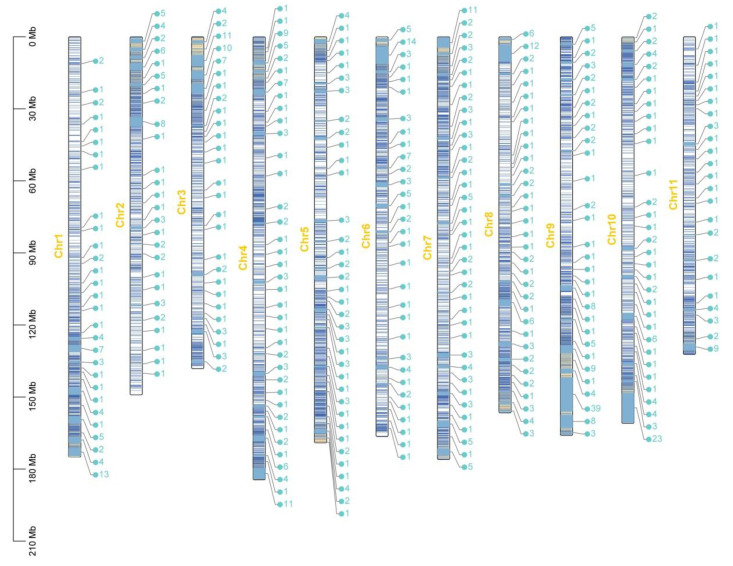
Physical map of the *CsOGs* on each chromosome of the coriander genome. The physical map of the *CsOGs* was constructed based on the chromosomal position of each gene, starting from the beginning of each chromosome. Chr1–Chr11 represents the 11 chromosomes of the coriander genome. The black oblongs indicate different chromosomes. The numbers on the right side of each chromosome indicate the number of *CsOGs*, while lines within black oblongs represent the chromosomal positions of each *CsOG*. Genes located on scaffold are not included in this figure.

The gene numbers and percentage of *ASGs* and *CsOGs* were calculated, and physical maps of *CsOGs* across 11 chromosomes were constructed on the basis of the *C. sativum* genome (Figure 3A and Figure 4). *CsOGs* were evenly distributed among the chromosomes, with the highest concentrations of *CsOGs* (3.4%) and *ASGs* (5.02%) observed on chromosome A11. Spearman’s correlation analysis indicated a significant positive relationship between the number of *CsOGs* on each chromosome and chromosome length (*p* = 0.000000003, *r* = 0.926). A similar correlation was observed for *ASGs* after excluding scaffold-associated genes. Thus, the longer chromosomes contained more *ASGs* or *CsOGs*. The intron–exon structure of the *CsOGs* and *ASGs* was further examined by categorizing genes into six groups based on the number of introns: intron-less, one intron, two introns, three introns, four introns, and five or more introns per gene (Figure 3B). The results revealed that 30.07% of the *CsOGs* and 25.12% of the *ASGs* were intron-less, while 38.79% of the *CsOGs* and 37.75% of the *ASGs* had one intron. The proportions of genes with two introns were 15.94% for *CsOGs* and 20.18% for *ASGs*, with fewer than 16% of *CsOGs* and 17% of *ASGs* falling into the other intron categories. The percentage of different types of intron number of *ASGs* and *CsOGs* were higher than that in *ECGs* (Table S1). Analysis of coding potential revealed that the percentage of coding genes within *CsOGs* and *ASGs* was lower than that of non-coding genes (Figure S1). Specifically, coding genes accounted for 19.23% of the *CsOGs* and 46.15% of the *ASGs*, while non-coding genes accounted for 80.77% of the *CsOGs* and 53.85% of the *ASGs*. These findings suggest that *CsOGs* and *ASGs* belong to distinct gene sets compared to *ECGs*.

### 2.3. Expression Patterns During Different Growth Stages and in Different Tissues

**Figure 5:**
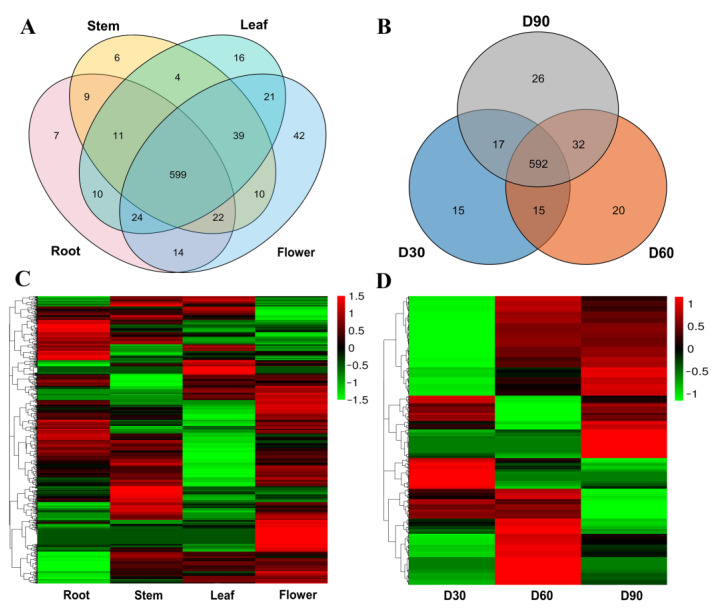
Expression patterns of *CsOGs* across different tissues and growth stages of coriander (*C. sativum*). (**A**) Venn diagram showing the distribution of 941 *CsOGs* across different tissues of *C. sativum*. (**B**) Venn diagram showing the distribution of 941 *CsOGs* across different growth stages of *C. sativum*. (**C**) Heat map of *CsOG* expression (FPKM > 0) across different tissues of *C. sativum*. The columns represent different tissues, with FPKM values indicating expression changes. (**D**) Heat map of *CsOG* expression (FPKM > 0) across different growth stages of *C. sativum*. The columns represent different growth stages, with FPKM values indicating expression changes. The red color in the heat map indicates high expression, while the green color indicates low expression.

Gene expression patterns often provide insights into gene functions [37]. The expression patterns of the *CsOGs* were analyzed to predict their potential functions. The protein sequences of 941 *CsOGs* were blasted with *C. sativum* ESTs and PUTs using TBLASTN (E-value cut-off: 1 × 10^−3^; query coverage and recognition rate: 70%). Considering the incomplete nature of PUTs and ESTs, transcriptome sequencing data from different growth stages and tissues were obtained from a previous study [23]. These datasets were subsequently used to analyze the expression patterns of the *CsOGs* (Figure 5). The RNA-Seq data confirmed that 834 *CsOGs* were expressed (Fragments Per Kilobase of exon model per Million mapped fragments (FPKM) > 0 in at least one tissue), with 44 *CsOGs* having functional annotations. Specifically, one gene was annotated in the KEGG database, five in the IPRSCAN database, and eleven in the NCBI Nrdb (Figure S2). Moreover, seventy-one *CsOGs* presented unique expression patterns: seven in roots, six in stems, sixteen in leaves, and forty-two in flowers (Figure 5A,C). In terms of growth stages, 61 *CsOGs* were uniquely expressed: 15 in the CS30 stage, 20 in the CS60 stage, and 26 in the CS90 stage (Figure 5B,D). Five genes (*Cs01G02001*, *Cs05G02239*, *Cs07G00515*, *Cs08G00942*, and *Cs11G00260*) were specifically expressed across different tissues (Table S2) or growth stages (Table S3), suggesting that *CsOGs* may play distinct roles in different growth stages and tissues.

**Figure 6:**
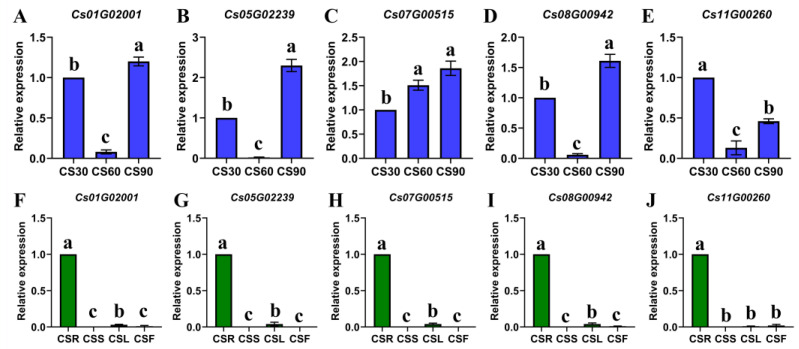
Expression levels of *CsOGs* across different tissues and growth stages of coriander (*C. sativum*). (**A**–**E**) Expression levels of *CsOGs* at different growth stages of coriander, with CS30, CS60, and CS90 representing leaves at 30 days, 60 days, and 90 days, respectively. (**F**–**J**) Expression levels of *CsOGs* in various tissues of coriander, with CSR, CSS, CSL, and CSF representing the roots, stems, leaves, and flowers, respectively. Y-axis indicates the relative expression level of standardized genes, and the qRT-PCR reactions were normalized using the *Actin* gene as reference for all comparisons. The data are presented as means ± SE from three independent measurements. Significant differences, as determined by one-way ANOVA, are indicated by different letters above the bars (*p* < 0.05).

The expression patterns of five *CsOGs* across three growth stages (CS30, CS60, and CS90) and four tissues (flower, leaf, stem, and root) were further analyzed via qRT-PCR. The results revealed that these *CsOGs* presented low expression levels across different growth stages (Figure 6A–E). In the tissue-specific analysis, *Cs11G00260*, *Cs08G00942*, and *Cs01G02001* were highly expressed in roots, had low expression in the leaves and flowers, and were not expressed in the stems (Figure 6F–J). Similarly, *Cs05G02239* and *Cs07G00515* were highly expressed in the roots, showed low expression in the leaves, and were not expressed in the stems or flowers. In conclusion, the expression analysis of these five *CsOGs* showed general or specific patterns in different tissues and developmental stages.

### 2.4. Expression Patterns of CsOGs in C. sativum After Cold Stress

**Figure 7:**
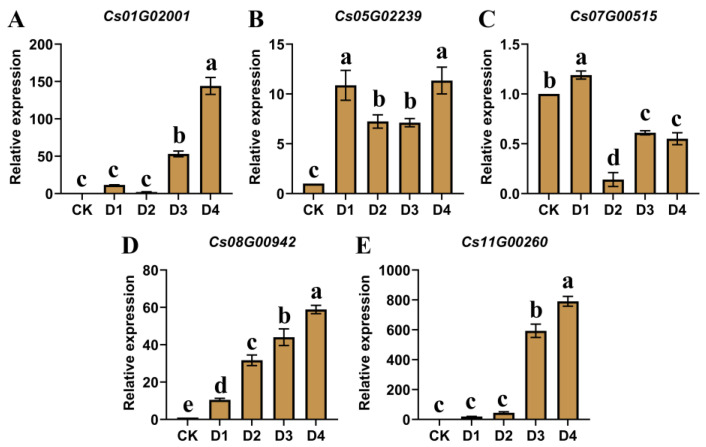
Expression patterns of five *CsOGs* in coriander (*C. sativum*) leaves after cold stress. (**A**–**E**) Expression levels of five *CsOGs* under cold stress. D1, D2, D3, and D4 represent 55-day-old *C. sativum* plants exposed to 4 °C, with leaf samples collected at 1 h intervals. Y-axis indicates the relative expression level of standardized genes, and the qRT-PCR reactions were normalized using the *Actin* gene as reference for all comparisons. The data are presented as means ± SE from three independent measurements. Significant differences, as determined by one-way ANOVA, are indicated by different letters above the bars (*p* < 0.05).

*OGs* are frequently associated with responses to biotic and abiotic stresses and often participate in stress–response signal transduction pathways [2,5]. Previous studies have shown that cold stress adversely affects coriander growth [28]. Therefore, it was hypothesized that *CsOGs* may play an important role in the response of leaves to cold stress. To test this hypothesis, 55-day-old *C. sativum* plants were exposed to 4 °C, and leaf samples were collected hourly (D1 to D4). The expression patterns of five *CsOGs* in leaves following cold stress were analyzed using qRT-PCR (Figure 7). Results indicated that the expression levels of *Cs01G02001*, *Cs05G02239*, *Cs08G00942*, and *Cs11G00260* were significantly upregulated compared to the control group. Notably, *Cs11G00260* had a 700-fold increase under cold stress compared with that under normal conditions. In contrast, *Cs07G00515* showed a significant decrease in expression in response to cold stress. These findings suggest that the five *CsOGs* may play roles in the cold stress response of *C. sativum*, potentially contributing to cold stress resistance mechanisms.

## 3. Discussion

A total of 38,508 *ECGs*, 1298 *ASGs*, and 941 *CsOGs* were identified in the *C. sativum* genome via stringent BLAST search criteria (Figure 1 and Figure 2). The analysis incorporated genomes from Phytozome v13, PUTs from plantGDB, and comparisons with databases such as UniProt-KB and Nrdb, Nr/Nt, and ESTs in NCBI. In previous studies, 165 *Arabidopsis*-specific genes and 1324 lineage-specific genes under TAIR7 and TAIR8 releases were identified, respectively [34,38]. These findings highlight how advancements in genome databases and the release of new plant genomes improve the accuracy of *OG* identification. The current study used an updated *C. sativum* genome containing 40,747 genes, improving the screening of *CsOGs* and *ASGs*. Although the potential for false positives exists, this approach represents an important step in refining *OG* identification within *C. sativum*. Previous research, with a BLAST E-value cut-off < 1 × 10^−1^, identified 165 *Arabidopsis*-specific genes [38]. In comparison, the more stringent criteria used in this study led to a greater number of *CsOGs*. Earlier studies may have missed potential positive *OGs* because of the use of less stringent criteria. By using multiple databases and stricter parameters, this study was able to identify a greater number of *OGs* than earlier research.

The results indicated that *OGs* generally have fewer exons and a lower proportion of multi-exon genes, making *CsOGs* and *ASGs* shorter than *ECGs* (Table 1, Figure 3B, and Table S1), which is consistent with previous reports [2]. The GC content of the *CsOGs* and *ASGs* did not significantly differ from that of the *ECGs*, which contrasts with observations in *Populus trichocarpa* [36] and *A. thaliana* [34]. Moreover, the proportion of intron-less genes was significantly higher in the *CsOGs* (30.07%) and *ASGs* (25.12%) than in the *ECGs* (19.91%) (Figure 3B and Table S1). The increased proportion of intron-less genes in *CsOGs* and *ASGs* may result from recent lineage-specific amplification, potentially driven by retro-transposition, which contributes to new gene creation [2,37]. Furthermore, intron-less genes may play a crucial role in biodiversity during species evolution [2,39]. Studies suggest that gene emergence and gene loss reflect a dynamic balance in gene evolution, where the addition of introns helps retain some *OGs* while others are lost over time [40]. However, further studies are needed to verify these evolutionary mechanisms. Chromosome distribution analysis revealed that 2.14% of *CsOGs* and 3.32% of the *ASGs* remained unannotated; thus, we hypothesized that these sequences may represent genuine genes rather than *C. sativum* genome annotation artifacts. These findings align with observations in sweet orange [37] and *B. rapa* [2]. Moreover, the highest concentrations of *CsOGs* and *ASGs* were observed on chromosome A11, and this may be related to the fact that longer chromosomes contain more genes, which is similar to a previous finding [2]. In conclusion, the *CsOGs* and *ASGs* in *C. sativum* appear to have undergone more rapid evolution compared with the *ECGs*.

Gene expression patterns are often associated with gene function, making them useful predictors of potential gene roles [2]. In this study, the expression patterns of 941 *CsOGs* were preliminarily assessed using RNA-Seq data. The analysis revealed that most *CsOGs* presented low expression levels in coriander, similar to observations in *A. thaliana* [4]. The comparison of genes expressed across growth stages and tissues identified five genes (*Cs01G02001*, *Cs05G02239*, *Cs07G00515*, *Cs08G00942*, and *Cs11G00260*) with specific expression patterns across both conditions. Further validation using qRT-PCR revealed that these five genes presented low expression levels across different growth stages (Figure 6A–E). Tissue-specific analysis indicated that five genes possess much lower expression levels in leaves, flowers, and stems than in roots (Figure 6F–J). Notably, the qRT-PCR results differed from the RNA-Seq findings (Tables S2 and S3), which initially suggested the specific expression of these genes across growth stages and tissues. This discrepancy may be attributed to differences in coriander varieties, sample collection times, or the limited and conditional expression of *CsOGs*. Additionally, the low expression levels of *CsOGs* may have hindered detection using conventional transcriptional analysis methods [34].

Studies have shown that *OGs*, which may be involved in stress–response signal transduction pathways, are often associated with biotic and abiotic stresses [5,8,41,42]. Given previous findings that cold stress impacts growth, yield, and metabolism in coriander [28], *C. sativum* plants were treated at 4 °C, and leaf samples were collected hourly (D1-D4) to explore the potential role of five genes under cold stress using qRT-PCR. The expression of these genes significantly changed in response to cold stress, particularly that of *Cs11G00260*, whose expression increased 700-fold compared with that under normal temperature conditions (Figure 7). These results suggest that *Cs01G02001*, *Cs05G02239*, *Cs07G00515*, *Cs08G00942*, and *Cs11G00260* may play a role in the cold stress response in coriander. A previous study showed that the *OG PpARDT* increases the abscisic acid (ABA) response in *Physcomitrium patens*, contributing to drought resistance [43]. In cowpea, the drought-induced gene *UP12_8740* showed significant differences in root expression under drought stress and increased drought resistance in transgenic plants [12]. In *Arabidopsis*, 1007 genes with unknown functions were analyzed for their response to various abiotic stresses, such as hypoxia, cold, salt, heat, osmotic, and oxidation stress, with 832 mutants showing sensitivity to treatments, indicating potential roles in signal transduction or adaptation mechanisms [14,44]. Cold stress also inhibited *OG BR2* expression and accelerated bolting and flowering in Chinese cabbage [19]. High stress-specificity has been highlighted as a characteristic associated with *OGs* [4,45,46]. Therefore, these research results on the response of *OGs* to stress strongly support the findings of this study.

Although the use of multiple databases and stricter parameters allowed for the identification of more *OGs* than earlier research did, some *CsOGs* may be eliminated as the number of genome-sequenced species in Apiaceae and the genus *Coriandrum* increases. The expression levels of many *CsOGs* may not be comprehensive by traditional transcription analysis methods, resulting in many *CsOGs* not being screened, and some important *CsOGs* may be missed. *CsOGs* respond to cold stress, but whether they are affected by other stresses is still unclear, and the molecular mechanism of their participation in cold stress needs to be thoroughly studied. Therefore, the next step of this study is to expand the screening conditions on the basis of RNA-Seq data, obtain more important *CsOGs*, and continue to study the molecular mechanism of these five genes involved in cold stress further.

## 4. Materials and Methods

### 4.1. Genome Datasets

The proteomic sequence of *C. sativum* was retrieved from the Coriander Genome Database (CGDB, http://cgdb.bio2db.com/) on 10 February 2022. A total of 124 complete genomes, including 122 non-Apiaceae genomes and two Apiaceae genomes (excluding *Coriandrum* species), were downloaded from Phytozome v13 (https://phytozome-next.jgi.doe.gov/) and CGDB for the identification of *ECGs*, *ASGs*, and *CsOGs* on 11 March 2022. The Plant Genome Database (PlantGDB) (http://www.plantgdb.org/prj/ESTCluster/progress.php) was used to retrieve PlantGDB-generated unique transcripts (PUTs) that were downloaded on 13 April 2022. The UniProt knowledgebase (UniProt-KB) was accessed from the Universal protein resource (UniProt) (http://ftp.ebi.ac.uk/pub/databases/uniprot/knowledgebase/) on 18 April 2022. Other databases, including expressed sequence tags (ESTs), nucleotide collection (Nr/Nt), and the Non-redundant protein database (Nrdb), were analyzed via the National Center for Biotechnology Information (NCBI) (https://www.ncbi.nlm.nih.gov/) on 15 June 2022.

### 4.2. Homology Search

The homology search was based on previous methods [2], screening three gene sets in coriander with an E-value cut-off of 1 × 10^−3^. BLASTP, TBLASTN, and BLASTN were used as the sequence alignment tools, and manual inspection of the resulting alignments was performed throughout the homology search. The genes were categorized into *CsOGs*, *ASGs*, and *ECGs*. *CsOGs* included genes with no homologs in non-*Coriandrum* species, *ASGs* contained genes with at least one homolog in Apiaceae species (excluding *Coriandrum*), and *ECGs* had at least one homolog outside the Apiaceae family.

### 4.3. Genic Features and Physical Mapping

Complete genome data for *C. sativum* were obtained from the CGDB. The length of protein, CDS, gene, and intron, the GC content of CDS, intron, and gene, and the number of exons per gene of *CsOGs*, *ASGs*, and *ECGs* were analyzed. The significant differences in different genic features among three gene sets were identified using one-way ANOVA. Chromosome localization data were extracted from chromosome sequences. Spearman’s correlation analysis was performed to assess the relationships between the percentages of *CsOGs* and *ASGs* on each chromosome and chromosome length. The physical map was constructed via R.

### 4.4. Gene Functional Annotation and Expression Evidence

The protein sequences of the *CsOGs* were compared with the ESTs and PUTs via TBLASTN, following the methods described in a previous study to confirm the expression data of the *CsOGs* [2]. RNA-Seq data from four *C. sativum* tissues (flowers, leaves, stems, and roots) and three growth stages (CS30, CS60, and CS90) were downloaded from the Genome Sequence Archive (GSA, https://ngdc.cncb.ac.cn/gsa/) under accession numbers CRA001658 and CRA001656 on 25 June 2022, respectively [23]. The analysis of the RNA-Seq data was performed according to previous methods [2,17,19]. The drawing of heat maps referred to previous studies [2,17].

### 4.5. Sampling and Expression Analysis

The *C. sativum* inbred line ‘NAXC3’ was provided by the vegetable research group of the School of Agriculture, Jilin Agricultural Science and Technology University, China. For different growth stages, leaves were collected at 30, 60, and 90 days (CS30, CS60, and CS90, respectively), with three biological replicates of three plants each. For tissue-specific analysis, roots, stems, leaves, and flowers were sampled at 85 days (CSR, CSS, CSL, and CSF, respectively), with three biological replicates of three plants each. The cold stress treatment involved exposure to 4 °C. Leaves weighing 1 g were obtained from 55-day-old coriander plants that were grown in nutrient bowls at 4 °C; CK, D1, D2, D3, and D4 represent exposure to 4 °C for 0 h, 1 h, 2 h, 3 h, and 4 h, respectively (three biological replicates, three plants per replicate). Coriander about 50-day-old showed higher physiological activity, which can more clearly reflect the effect of stress treatment [29,47,48]. All the samples were immediately frozen in liquid nitrogen and stored at −80 °C. Plant total RNA extraction, first-strand cDNA synthesis, and qRT-PCR were conducted according to previously described methods [17], and expression levels were calculated using the 2^−ΔΔCt^ method [49]. The primers used in this study are listed in Table S4.

### 4.6. Statistical Analysis

The qRT-PCR data were analyzed using one-way ANOVA in IBM SPSS Software v26 (Chicago, IL, USA). Results are presented as means ± SE. Spearman’s correlation analysis was performed to calculate the relationship between the number of *CsOGs* on each chromosome and chromosome length via the formula in Microsoft Excel v2010 (Redmond, WA, USA).

## 5. Conclusions

In this study, a total of 1298 *ASGs* and 941 *CsOGs* were identified in coriander genome. Compared with the *ECGs*, the *CsOGs* and *ASGs* presented a greater GC content, shorter gene lengths, and fewer multi-exon genes. *ASGs* and *CsOGs* were evenly distributed across 11 chromosomes, with the highest concentrations observed on chromosome A11. RNA-Seq analysis revealed five *CsOGs* with specific expression patterns across various tissues and growth stages. qRT-PCR analysis revealed that these five *CsOGs* generally presented specific patterns under normal conditions, but their expression significantly increased under cold stress. In particular, the expression of *Cs11G00260* increased 700-fold under cold stress conditions, suggesting that *Cs11G00260* may play a certain role in the cold stress response. These findings provide valuable insights into the function of *CsOGs* in coriander, laying the groundwork for further functional analyses.

## Figures and Tables

**Table 1:** Genic features of the *CsOGs*, *ASGs*, and *ECGs*.

Features	941 *CsOGs*	1298 *ASGs*	38,508 *ECGs*
	Mean (SD)	Mean (SD)	Mean (SD)
Protein length	115.72 (52.80)	171.00 (102.65)	371.21 (281.89)
CDS length	350.04 (158.40)	515.93 (308.00)	1116.60 (845.67)
Gene length	1778.90 (3229.96)	1666.62 (2721.17)	3729.82 (4891.99)
Intron length	646.41 (1385.30)	583.73 (2157.20)	536.15 (1305.61)
CDS GC (%)	42.59 (5.54)	42.79 (5.04)	42.69 (3.26)
Intron GC (%)	23.44 (16.15)	24.71 (15.12)	25.69 (13.20)
Gene GC (%)	37.64 (5.15)	38.64 (5.04)	37.22 (3.64)
Exons/gene	2.30 (1.42)	2.42 (1.37)	4.98 (4.71)

## Data Availability

All data supporting the findings of this study are available within the paper and within its Supplementary Materials published online.

## Supplementary Materials

The following supporting information can be downloaded at https://www.mdpi.com/article/10.3390/plants14050778/s1, Figure S1: Coding capability analysis of CsOGs and ASGs. The Y-axis marks the percentage of coding and non-coding. The purple bar chart shows the percentage of coding; the green bar chart shows the percentage of non-coding. Figure S2: Analysis of specific gene annotation. KEGG indicates Kyoto Encyclopedia of Genes and Genomes. IPRSCAN indicates a non-redundant database, integrating domains, functional sites, and protein families. Swissprot indicates a high-quality, manually annotated, non-redundant protein sequence database. Nr indicates a non-redundant database. Table S1: Gene numbers and percentage of different types of intron number of ECGs. Table S2: FPKM values of CsOGs in different tissues of coriander. Table S3: FPKM values of CsOGs in different growth stages of coriander. Table S4: Primers used for qRT-PCR analysis.

## Abbreviations

The following abbreviations are used in this manuscript:

ABA	Abscisic acid
*ASGs*	Apiaceae-specific genes
CGDB	Coriander Genome Database
CsOGs	*C. sativum OGs*
*ECGs*	Evolutionarily conserved genes
ESTs	Expressed sequence tags
FPKM	Fragments Per Kilobase of exon model per Million mapped fragments
GSA	Genome Sequence Archive
KEGG	Kyoto Encyclopedia of Genes and Genomes
NCBI	National Center for Biotechnology Information
Nrdb	Non-redundant protein database
Nr/Nt	Nucleotide collection
*OGs*	Orphan genes
PUTs	PlantGDB-generated unique transcripts
SUS	Sucrose synthase
TEs	Transposable elements
TFs	Transcription factors
UniProt	Universal protein resource
UniProt-KB	UniProt knowledgebase
