# Supplementary material for: Genome-Wide Identification, Characterization, and Expression Analysis of Orphan Genes Within Coriander
Source: Plants (Basel). 2025 Mar 3;14(5):778. doi: 10.3390/plants14050778 (PMC11901849; doi:10.3390/plants14050778)
Supplement: Supplementary file 1 [file plants-14-00778-s001.zip › plants-3485798-Supplementary Figures.pdf]

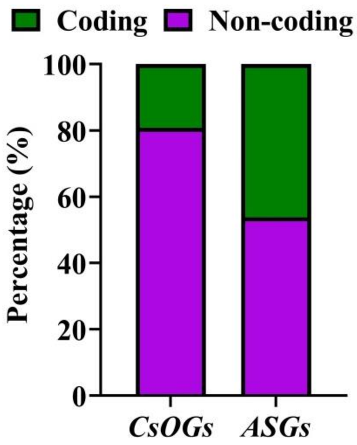

Figure S1. Coding capability analysis of *CsOGs* and *ASGs*. The Y-axis marks the percentage of conding and non-coding. The purple bar chart shows the percentage of coding, the green bar chart shows the percentage of non-coding.

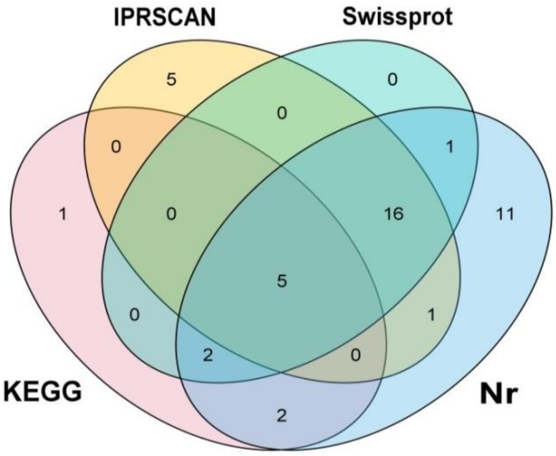

Figure S2. Analysis of specific gene annotation. KEGG indicates Kyoto Encyclopedia of Genes and Genomes. IPRSCAN indicates a non-redundant database integrating domains, functional sites, and protein families. Swissprot indicates a high-quality, manually annotated, non-redundant protein sequence database. Nr indicates non-redundant database.
